# Isolated diffuse invasive renal aspergillosis in an immunocompromized patient due to longstanding steroid treatment: a case report

**DOI:** 10.4076/1757-1626-2-6825

**Published:** 2009-07-16

**Authors:** Aleksandar Vodovnik

**Affiliations:** Department of Cellular Pathology, Medway Maritime HospitalGillingham, ME7 5NYUK

## Abstract

A 53-year-old Indian lady suffered from type 2 diabetes and hypothyroidism and was on longstanding steroid therapy. She was urgently admitted to the hospital with a high white cell count and high creatinine. On imaging no space occupying lesions were shown. In spite of intensive therapy the patient died a week after admission. Post mortem examination revealed markedly enlarged kidneys with areas of necrosis, hemorrhagic infarction, inflammatory response and granulomas related to the widespread glomerular, tubulo-interstitial and vascular involvement by aspergillus. Renal disease may present as bilateral diffuse parenchymal involvement with blood vessel invasion causing organ failure.

## Introduction

Aspergillus species have angioinvasive properties and frequently disseminate from the lung to a variety of organs via hematogenous spread. This is almost exclusively seen in patients with an impaired immunity and mortality is significant, varying from 30-95% [1-4]. Renal involvement is uncommon and often associated with the formation of abscesses presenting as space occupying lesions on imaging [5,6]. A case of isolated diffuse invasive renal aspergillosis in an immunocompromized patient due to longstanding steroid treatment is presented.

## Case presentation

The patient was a 53-year-old Indian lady who suffered from type 2 diabetes and hypothyroidism, which were controlled by Insulin and Metformin, and Thyroxine, respectively. She was a vegetarian, non-smoker and non-alcoholic. Her husband was treated for an active tuberculosis in his thirties. The patient suffered fevers of unknown origin a year ago but tuberculosis was never proven. Although the patient showed no active symptoms of SLE, she had a raised ESR, ANA and antibodies to double-stranded DNA on several occasions. The patient was on longstanding steroid therapy. On return from a prolonged visit to India, she became violently ill and was admitted to the hospital as confused, vomiting and lethargic. Laboratory tests showed a high white cell count and high creatinine. Treatment included hemofiltration, steroids, cyclophosphamide and plasma exchange. CT of the head showed a normal ventricular system, moderate cerebral atrophy and no focal intracranial lesions. On the chest X-ray, a wide spread, ill-defined shadowing in both lung mid zones was noted; the appearences on the left were also suggestive of consolidation. The abdominal US revealed a normoechogenic renal tissue on both sides with no evidence of hydronephrosis. The patient deteriorated and died a week after admission. A coroner’s post mortem examination was requested. Autopsy tissue samples were processed and prepared by steadandard histological techniques. Selected samples from the kidney were additionally stained by PAS and Gomori’s methenamine silver stains.

**Figure 1.**
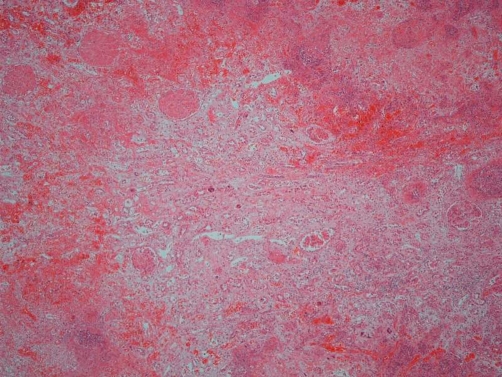
Haemorrhagic tissue infarction and necrosis of the kidney (Hematoxylin and Eosin, 20X).

**Figure 2.**
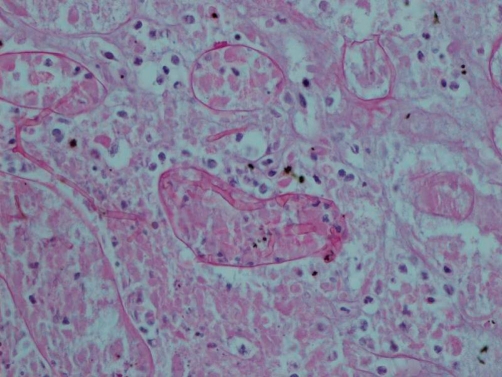
Renal tubuli containing Aspergillus hyphae (PAS, 100X).

**Figure 3.**
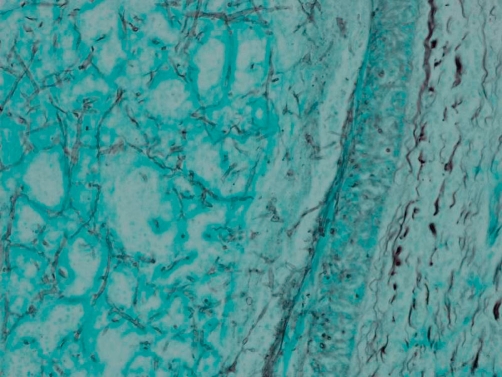
Renal angioinvasion by Aspergillus hyphae (Gomori methenamine silver, 100X).

**Figure 4.**
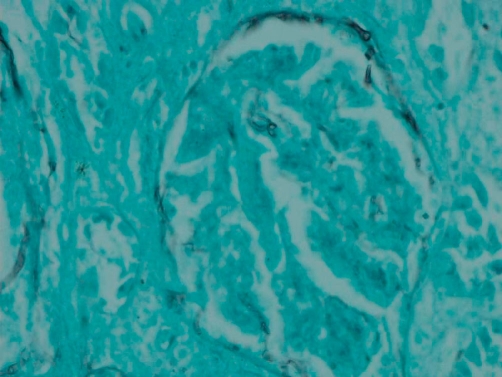
Renal glomerular involvement by Aspergillus hyphae (Gomori methenamine silver, 100X).

**Figure 5.**
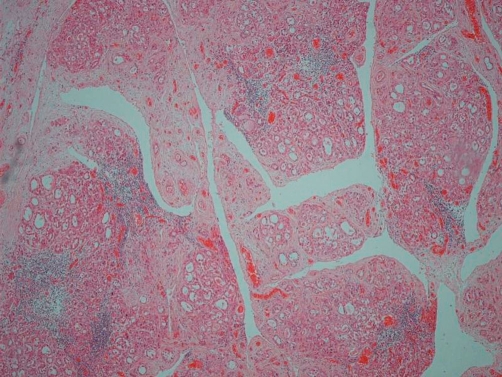
Thyroid nodular lymphoid stromal infiltration and oxyphilic change of the follicular epithelium (Hematoxylin and Eosin, 20X).

Post mortem examination revealed markedly enlarged kidneys showing geographical areas of necrosis. The lungs showed focal granular yellow-grey areas of consolidation. The thyroid was firm and nodular. Microscopic examination of the kidneys showed areas of necrosis, hemorrhagic infarction, and a mixed inflammatory infiltrate with granulomas related to the widespread glomerular, tubulo-interstitial and vascular involvement by aspergillus hyphae (Figures 1-4). There was extensive peri-renal spread of the fungus and associated inflammatory response. The lungs showed a bronchopneumonia. Hashimoto thyroiditis was observed (Figure 5). An extensive tissue search failed to find aspergillus in any other organ except the kidney. The rest of the urinary tract was also free of aspergillus.

## Conclusions

Invasive renal aspergillosis is a rare disease with only a few cases reported in the literature [1,2]. It is usually associated with AIDS and organ transplantation [5,6]. Although the point of entry is often the respiratory tract, in isolated cases of extra-pulmonary involvement it remains unclear. The diagnostic options in invasive aspergillosis are relatively few. Abscesses or infarctions may be recognized on US and CT. The abscess may be a subject to percutaneous aspiration and drainage, which in turn can serve as a useful diagnostic tool. Although hematuria, pyuria and proteinuria are frequent, Aspergillus cultures are typically negative. A retrograde pyelography and intravenous pyelogram may be helpful when dealing with a localized lesion. The presence of precipitating antibodies to Aspergillus species has been reported, although its diagnostic value remains unclear [1,7]. As highligted in this case, a renal disease may present as bilateral diffuse parenchymal involvement with blood vessel invasion causing the organ failure. Awareness of this possibility and early attempts at diagnosis and treatment are therefore of crucial importance, both proven to be a major challenge. When high-risk patients develop a compatible clinical picture, empiric treatment should be initiated as diagnostic testing is undertaken. An intravenous antifungal therapy is required. Voriconazole is now considered the drug of choice for invasive aspergillosis because of better tolerance and improved survival comparing with amphotericin [7].

## Abbreviations

AIDS: acquired immunodeficiency syndrome
ANA: antinuclear antibody
CT: computerized tomography
DNA: deoxyribonucleic acid
ESR: erythrocyte sedimentation rate
PAS: periodic acid Schiff
SLE: systemic lupus erythematosus
US: ultrasound
